# DNA and Protein Analyses to Confirm the Absence of Cross-Contamination and Support the Clinical Reliability of Extensively Hydrolysed Diets for Adverse Food Reaction-Pets

**DOI:** 10.3390/vetsci5030063

**Published:** 2018-06-26

**Authors:** Isabelle Lesponne, Jérôme Naar, Sébastien Planchon, Tommaso Serchi, Mauricio Montano

**Affiliations:** 1 Research & Development, Royal Canin SAS, 30470 Aimargues, France; jerome.naar@royalcanin.com; 2 Environmental Research and Innovation Department, Luxembourg Institute of Science and Technology, Belval, 4008 Luxembourg; sebastien.planchon@list.lu (S.P.); tommaso.serchi@list.lu (T.S.); 3 Mars Petcare Central Laboratory, Mars Inc., 30470 Aimargues, France; mauricio.montano@effem.com

**Keywords:** AFR, food allergy, pet food, nutrition, hydrolysed protein, DNA, cross contaminations, dog, cat, ancillary protein, food purity

## Abstract

Adverse food reactions (AFR) are a common cause of skin diseases in cats and dogs. The correct diagnosis and management of AFR relies upon clinical nutrition. The reliability of commercial hypoallergenic diets commonly used in AFR has been questioned because studies have shown the presence of proteins not declared on the label ingredients. It is proposed that extensively hydrolysed protein-based diets constitute a reliable nutritional solution. Royal Canin Anallergenic™ Canine and Feline diets are formulated with very low molecular weight feather protein and purified corn starch. Protein gel electrophoresis and thin layer paper chromatography were used to characterize protein hydrolysis in these diets and their hydrolysed raw materials; protein species were identified by mass spectrometry. To detect cross-contaminating protein, species-specific DNA was measured and correlated with ancillary protein content using calibration curves. The only protein components detected in the extensively hydrolysed feather protein raw material were amino acids and small oligopeptides. GBSS-I (Granule-bound starch synthase 1) was detected in the finished diets; this has not been reported as a clinically apparent allergen in dogs or cats. The DNA threshold corresponding to the maximum acceptable level of ancillary protein was not exceeded in 99.9% of more than 2150 product batches tested and no products were released to the market with cross-contaminating proteins. These results demonstrate the extensive level of protein hydrolysis in Royal Canin Anallergenic™ Canine and Feline diets and the absence of cross-contaminating protein, both key requirements for a diet to be used during diagnosis and for management of pets with AFR.

## 1. Introduction

Skin diseases are one of the most frequent presenting complaints for cats and dogs in veterinary practice [1,2,3] and they are frequently caused by adverse food reactions, or AFR, which is the third most common skin allergy in dogs and the second most common skin allergy in cats [4]. The signs of moderate to severe cutaneous AFR (CAFR) are likely to substantially affect the overall quality of life of the pet. Signs include non-seasonal pruritus with or without associated skin lesions, such as erythema, lichenification and excoriations in dogs [5,6] and crusting and excoriations of the head and Neck in cats. AFR may also have digestive signs such as soft stools or diarrhoea, abdominal discomfort and, less frequently, vomiting [7].

The clinical signs of CAFR are not pathognomonic; the diagnosis must be established by the reduction or cessation of clinical signs when the animal is fed exclusively an elimination diet that excludes the suspected allergens (the elimination trial), followed by a relapse upon reintroducing the original diet or its suspected ingredient (the re-challenge) [8,9,10]. Commercial hydrolysed diets are a convenient option for elimination trials and the long-term maintenance feeding of pets with AFR; unlike some home-made diets, they are balanced, not time consuming for the owner and they do not need to be tailored to the dietary history of the individual.

The goal of hydrolysed protein diets is to help prevent the recognition of epitopes that potentially trigger an adverse food reaction. There is currently no consensus on the precise threshold in protein size for it to be considered truly hypoallergenic but specialists agree that protein should be hydrolysed as extensively as possible, providing that there are no issues with bitterness or osmotic diarrhoea. The extent of hydrolysis can be determined by measuring the molecular weight of proteins in the diet, with a single amino acid weighing between 70 and 250 Da. Protein may be considered to be partially hydrolysed, or extensively hydrolysed to the level of oligopeptides and free amino-acids, resulting in a final very low molecular weight protein hydrolysate. Most commercial pet diets currently marketed as ‘hypoallergenic’ are partially hydrolysed; it has been reported that 25–50% of dogs still presented with clinical signs when fed hydrolysed diets containing protein ingredients to which they were allergic when these were intact proteins [11]. For this reason, researchers have been interested in the production of very low molecular weight protein hydrolysates.

The absence of cross contamination in any elimination diet is also fundamental. Recently, the detection of undeclared ingredients and large proteins in some commercial AFR-diets has raised concerns, especially with respect to non-prescription diets [12,13]. Discrepancies have been found between protein analyses and food labelling in about 75% of tested diets in some studies [12,14,15]. In the 2018 critically appraised topic (CAT) review of 18 relevant studies, unlabelled ingredients were found in a median of 45% of all tested diets (between 33% and 83% in the novel/limited ingredients diets marketed for elimination trials) and ingredients stated on the label were found to be missing in a median of 1%. Only one instance of possible mislabelling was found for hydrolysate-based pet foods [16].

Royal Canin Anallergenic^TM^ Canine and Feline diets (Royal Canin^®^, Aimargues, France) are veterinary-prescribed diets formulated with extensively hydrolysed poultry feather as the protein source and purified corn starch as the carbohydrate source. The objectives of the studies presented here were:

1. To confirm the specific protein composition of these diets and the extensive level of hydrolysis of their feather protein source
2. To confirm the effectiveness of Royal Canin’s cross-contamination risk management process for these diets through a specially designed DNA-based testing strategy.

## 2. Material and Methods

### 2.1. Analyses of Protein Composition

#### 2.1.1. Protein Separation by Protein Gel Electrophoresis and Chromatography

The size of proteins in a range of diets and in some of their raw materials were assessed using protein gel electrophoresis (Table 1).

Samples were extracted according to a Hot-SDS protocol, using sodium dodecyl sulphate (SDS) and dithiothreitol (DTT) (supplier for both, Sigma Aldrich, Saint-Louis, MO, USA). Briefly, 100 mg of material were extracted with 1 mL of SDS buffer at 22 °C with agitation. Proteins in the supernatants were quantified using the Reducing agent Compatible Denaturing agents Compatible protein assay (RC DC™ Protein Assay: protein assay from Bio-Rad supplier, Hercules, CA, USA), which has been shown to be a simple and robust method compatible with many of the potentially interfering compounds in the extraction buffers (e.g., DTT and SDS). The method was based on a modified and improved version of the Lowry method [17]. Extracts corresponding to 20 µg protein were then separated using 12% bis-tricine gels. Gels were stained with Serva purple (supplier SERVA Electrophoresis GmbH, Heidelberg, Germany). Protein sizes were estimated using a molecular weight ladder (Precision Plus Protein Unstained Standards from Bio-Rad).

12% bis-Tricine gels were suitable for mid- and high-molecular-weight proteins. However, accurate and reproducible separation of proteins in the ultra-low molecular-weight range (below 2 kDa) was difficult by gel electrophoresis. Consequently, ascending thin-layer paper chromatography was tested as an alternative separation method. Volumes of 0.5 µL of each sample and a control solution of lysine 10 mM were deposited on filter paper (the stationary phase). After drying, the paper was placed vertically in a beaker with a 1 cm layer of 0.7% ammoniac/33% isopropanol/66% water (the mobile phase). When the mobile phase reached the top of the paper it was transferred for drying under a hood before being sprayed with a solution of 2% ninhydrin in ethanol [18,19]. Ninhydrin allows the visualization of oligopeptides and amino acids, becoming purple coloured on binding to the primary and secondary amine group of amino acids. Colour development is much more intense for single amino acids compared with peptides and intact proteins, for which the expected colour development, if any, is very low.

#### 2.1.2. Protein Identification by Mass-Spectrometry

The protein bands observed on the electrophoresis gels were cut out, trypsin digested and the proteins were identified precisely using MALDI Tof-Tof, (MALDI: Matrix-assisted laser desorption/ionization; TOF: Mass spectrometer with Time of Flight (TOF) detector; Trypsin Gold from Promega, Madison, WI, USA). This mass spectrometry technique is highly sensitive and provides rapid and rigorous analysis of complex samples, measuring the profile of all detectable peptide species over the full mass range. As it relies on the charge of the analytes, the peptides should contain at least one charged amino acid (lysine or arginine for a positive charge and aspartic or glutamic acid for a negative charge). In Tof-Tof mode, the selected peptides are fragmented by collision with an inert gas. A second analyser measures the masses of these fragments and the sequence of the original peptides can be derived via comparison with a worldwide protein identification database.

### 2.2. Analyses for the Detection of Cross-Contaminating Proteins

Because Anallergenic^TM^ diet formulae contain highly hydrolysed feather proteins, along with other raw materials, classical tests were insufficient to detect ancillary proteins. A new approach was developed to derive the levels of cross-contaminating proteins from their DNA concentrations.

#### 2.2.1. DNA to Protein Calibration

Calibration curves associating DNA concentration with protein levels were developed based on the measurement of species-specific DNA in product samples spiked with known and increasing amounts of ancillary proteins. The dried product was ground to approximately 1 mm with a Lab Mill 3600 (Perten Instruments, Stockholm, Sweden) and re-ground with a grinder ZM200 (Retsh GmbH, Haan, Germany) using a 0.75 mm mesh. DNA was extracted from 0.5 g of the ground product using the Sure-food^®^ Prep Basic kit (S1052, R-Biopharm AG, Darmstadt, Germany) according to the manufacturer’s instructions. DNA from the final extraction step was diluted in 100 μL of nuclease free water and was quantified by DNA intercalation fluorescence using the Quantifluor dsDNA kit (E2670, Promega, Madison, WI, USA). 

Table 2 shows the DNA concentrations corresponding to different ancillary proteins used to cross-contaminate diets to a level of 0.5% (as fed). This inclusion level, or NPPI (no protein pollution index %), was equivalent to the level of ancillary protein in the Anallergenic™ diet that had triggered no clinical signs when tested in a pilot clinical study [20] (Section 2.2.2).

#### 2.2.2. Analysis of Finished Product for Cross-Contaminating Proteins

There is currently no clear consensus on the maximum concentration of a cross-contaminating protein that can be present in an AFR diet without eliciting a reaction in a sensitized AFR-affected pet. The threshold used for Royal Canin Anallergenic™ Canine and Feline diets was taken from a multicentre pilot trial performed in Europe [20]. In that trial, dogs with complex AFR (i.e., AFR in combination with other disorders such as Canine Atopic Dermatitis) or refractory AFR (AFR managed with a standard hypoallergenic diet but not fully stabilized and requiring periodic medication) were fed a highly hydrolysed feather-based protein diet. All dogs had significant improvements in veterinary-assessed skin condition and there was a decrease in the average Global Skin Score (GSS) in 8 weeks (GSS is a combination of lesion scores by Canine Atopic Dermatitis Extent and Severity Index [CADESI] and pruritus scores) [21]). Given the clinical effectiveness of that diet, the 0.5% inclusion level of ancillary protein (NPPI 0.5%) that was found to be present was used to set the DNA conformity threshold for cross-contamination in the studies reported here. The lowest DNA concentration corresponding to a source-specific NPPI of 0.5% was 2.1 µg/g (Table 2). A security margin was applied to obtain a conformity threshold concentration of 1.2 µg/g for total DNA content, that is, the maximum concentration of total DNA that corresponded to an acceptable level of ancillary protein for an AFR diet.

**Figure 1:**
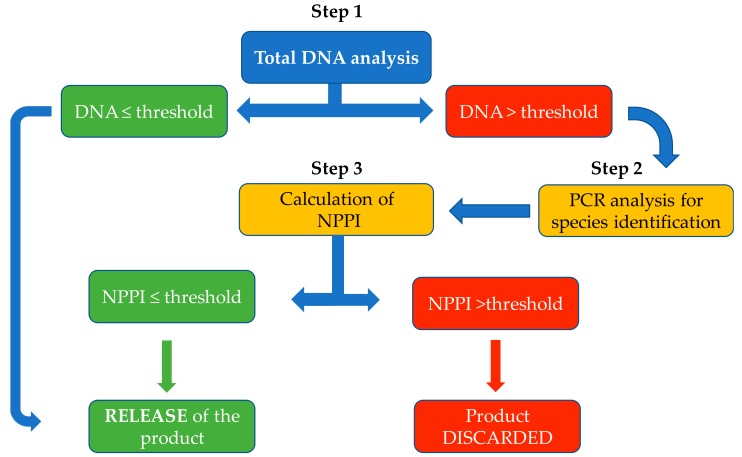
Decision tree of analytical steps used by Royal Canin for product validation of Anallergic™ extensively hydrolysed protein-based diets. NPPI (no protein pollution index) is the level of ancillary protein expressed as a percentage.

In order to verify the absence of cross-contaminating protein in Royal Canin Anallergenic™ Canine and Feline diets, production lots were tested by a 3-step DNA testing method (Figure 1).

- Step 1: The total content of DNA in the diets was measured by the method described above and compared with the DNA conformity threshold. The sample was considered to be within specification if the total measured DNA was ≤1.2 µg/g.
- Step 2: When the concentration of total DNA exceeded 1.2 µg/g, polymerase chain reaction (PCR) analyses were conducted to identify the source of the contamination. Species detection was carried out by targeted quantitative reverse transcriptase PCR conducted by an accredited laboratory.
- Step 3: The calibration curves were then used to determine the NPPI for the specific protein source. The NPPI was considered to be acceptable if it was ≤0.5% (corresponding to a DNA concentration for example of ≤4.4 µg/g for duck protein sources). In this way, the calibration curves allowed a more refined assessment of protein contamination in those samples that contained more than the threshold level of total DNA.

The full procedure is summarized in Figure 1 and this represents the actual product validation process used by Royal Canin in the commercial production of Anallergic™ diets. The same conformity threshold is used as described above and the procedures determine whether or not a production lot is released to the market.

## 3. Results

### 3.1. Protein Composition of the Diets

**Figure 2:**
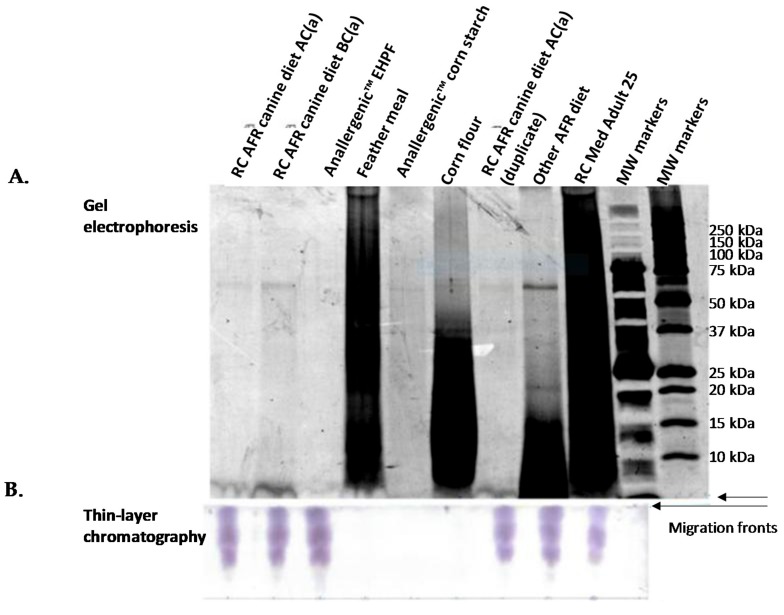
Protein analyses on diets and raw materials in parallel. (**A**) Protein electrophoresis of Anallergenic™ canine diet, after and before coating with palatability enhancer, of other commercial diets and of different feather and corn raw materials. The gel is a 12% bis-tricine gel with serva purple staining with the same quantity of protein loaded in each well. The migration of protein fragments is shown from the top of the figure to the bottom (migration front). (**B**) Thin layer chromatography to visualize the lowest molecular weight protein components. Separation of the fragments is primarily dependent upon their affinity for the mobile phase rather than molecular weight and the migration ascends from the bottom to the top of the figure. Separated oligopeptides and amino acids were visualized with ninhydrin staining. RC AFR AC(a) is Royal Canin Anallergenic™ canine diet after coating, batch (a); RC AFR BC(a) is Royal Canin Anallergenic™ canine diet before coating, batch (a); Anallergenic™ EHPF is extensively hydrolysed poultry feather used in Royal Canin Anallergenic™ diets; Other AFR diet is another commercial dry diet designed for dogs with adverse food reactions; RC Med Adult 25 is Royal Canin^©^ Medium adult kibble; MW is molecular weight.

**Figure 3:**
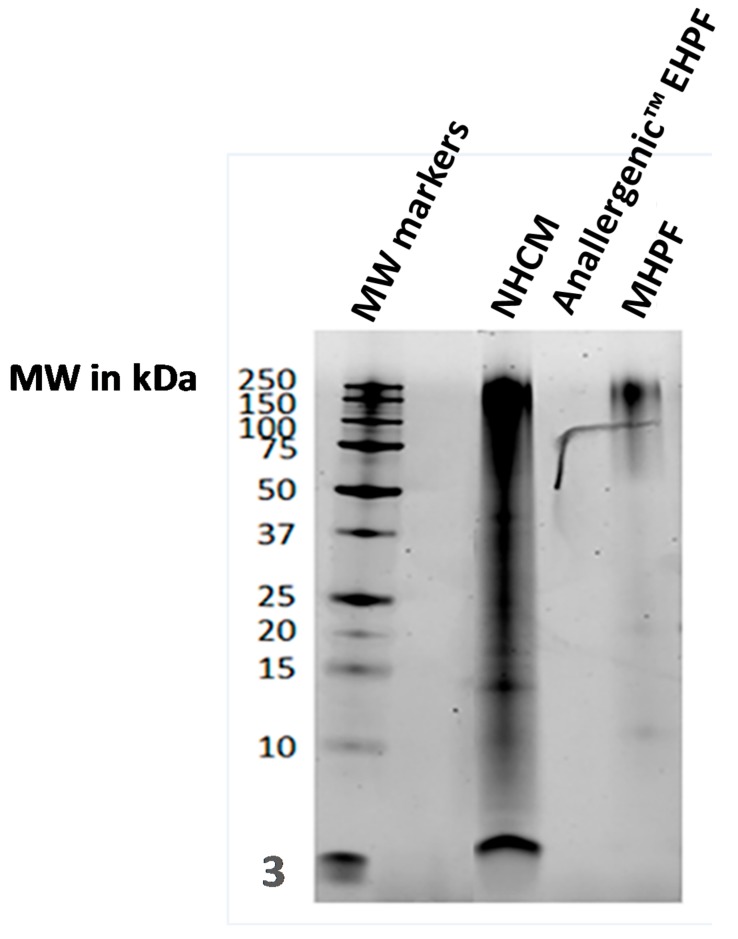
Protein electrophoresis on three different poultry raw materials, including the extensively hydrolysed feather protein of Anallergenic^TM^ diets. The same quantity of protein was loaded into each well of a 12% bis-tricine gel and separated proteins were stained with serva purple. NHCM is non-hydrolysed chicken meal; Anallergenic™ EHPF is extensively hydrolysed poultry feather used in Royal Canin Anallergenic™ diets; MHPF is mildly hydrolysed poultry feather. MW, molecular weight. Note that the artefact visible on the two columns on the right hand side was a deposit before migration.

There was a wide range of protein sizes in unhydrolysed feather meal (Figure 2). Protein sizes in hydrolysed feather raw materials differed according to the extent of hydrolysis (Anallergenic™ extensively hydrolysed poultry feather (EHPF) in Figure 2 and Figure 3 and mildly hydrolysed poultry feather (MHPF) in Figure 3) and large proteins were still present in the mildly hydrolysed feather protein source.

No protein bands were observed in bis-tricine electrophoresis gels for the extensively hydrolysed feather protein source in the Royal Canin Anallergenic™ canine diet (Anallergenic™ EHPF in Figure 2A and Figure 3). The only protein components detected for the Anallergenic™ EHPF raw material were amino acids and oligopeptides observed by the thin layer chromatography (Figure 2B). These small peptides were also present in all the diets tested (Figure 2). 

**Figure 4:**
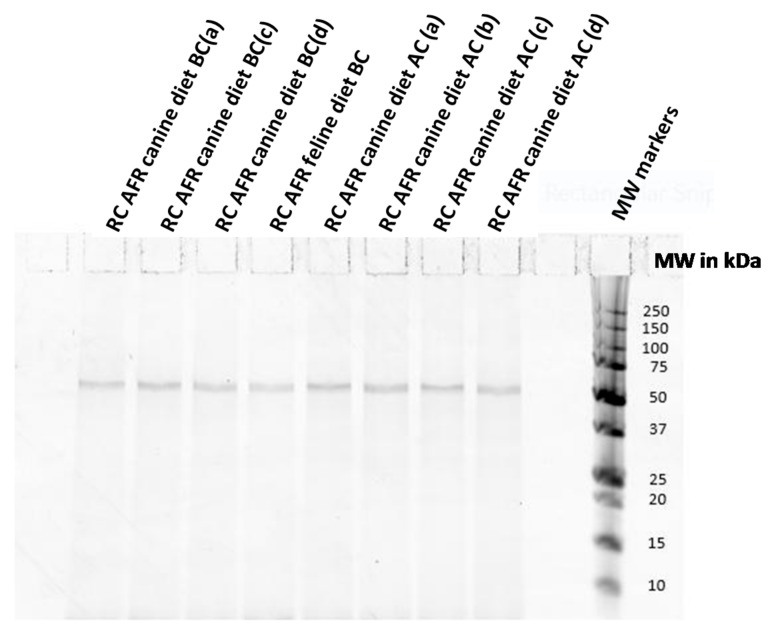
Protein electrophoresis of different production lots of Royal Canin Anallergenic™ canine and feline diets before and after coating with palatability enhancers. RC AFR diets are Royal Canin Anallergenic™ diets before coating (BC) or after coating (AC). a, b, c and d indicate different production batches. MW, molecular weight. Note that free amino-acids are not visualized by this technique.

**Figure 5:**
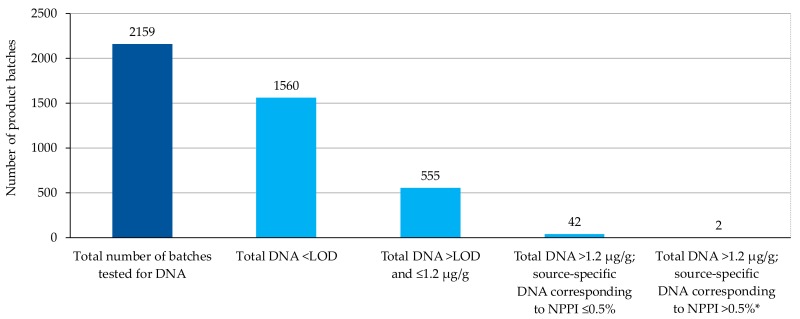
Results of DNA tests for all batches of canine and feline Anallergenic™ diets intended for commercial use (subject to being below the contamination threshold) from 2011 to April 2018. These did not include samples from equipment rinsing batches or batches from the preliminary steps of industrialization of production. More than 99.9% of the tested batches conformed to the contamination limits, having ancillary protein-related DNA content either not detectable (*n* = 1560, 72.3%), below the total DNA conformity threshold of 1.2 µg/g (*n* = 555, 25.7%), or above the total DNA conformity threshold but below a source-specific NPPI of 0.5% based on qRT-PCR and DNA-protein calibration curves (*n* = 42, 1.95%). In total, less than 0.1% of the tested batches (*n* = 2/2159) did not conform to the standards and were then discarded. LOD, limit of detection = 0.003 µg/g; NPPI, no protein pollution index; qRT-PCR, quantitative reverse transcriptase polymerase chain reaction. * Over the threshold specification used for maximum acceptable levels of ancillary protein, hence products were discarded.

Figure 4 shows that the extent of protein hydrolysis was the same in Royal Canin Anallergenic™ canine and feline diets from several production lots corresponding to different production periods. The protein band observed in all Anallergenic™ diets in Figure 5 corresponded to approximately 60 kDa and was identified by MALDI-TOF-TOF as granule-bound starch synthase 1 (GBSS-I) from Zea mays species. GBSS-I is a corn protein associated with carbohydrate metabolism that was previously identified in all hydrolysed diets tested in an in vitro study [13,22]. Corn lipid-transfer proteins (LTPs) were not detected in Royal Canin Anallergenic™ diets. The Zea m14 LTP is a major corn allergen found previously in corn flour but not in corn starch and having a reported size of 9–10 kDa [23]. There were no visible bands of this size for Anallergenic™ diets in the electrophoresis gels and no LTPs were identified by MALDI-TOF-TOF.

In summary, except for the hydrolysed feather protein source itself and for GBSS, the electrophoresis gels demonstrated the absence of ancillary protein in the finished Anallergenic™ products, indirectly confirming that there was no protein from either the palatability enhancers (aromas included in the coating steps of kibbles) or from included fats. There was indirect evidence for the repeatability of the results on the basis of identical findings from several production lots and different production periods.

### 3.2. Cross-Contamination Risk Management

DNA tests were performed on more than 2150 individual batches of Royal Canin Anallergenic™ canine and feline commercialized diets produced between 2011 and April 2018. In most cases, total DNA concentration was below the limit of detection (0.01% of finished product, which is standard for PCR tests) and most product batches were validated in Step 1 of the cross-contamination test procedure. In total, more than 99.9% of product batches were found to be within specification, with either a total DNA content ≤ 1.2 µg/g or a source-specific NPPI ≤ 0.5% (DNA threshold dependent on source) [24,25] (Figure 5). 

## 4. Discussion

The results presented on the protein-related DNA content of both canine and feline diets confirm the benefits of following strict criteria for all steps in the commercial production of AFF diets in order to ensure the absence of cross-contamination. Quality control encompasses the choice of raw materials, transportation of raw materials to the factories, plant adaptations, strict cleaning procedures and analyses confirming the absence of ancillary protein in the final product. As the absence of cross-contamination is key to the clinical effectiveness of ‘anallergenic’ or hypoallergenic diets, Royal Canin mandates that none of its Anallergenic™ diets can be sold in the market without having a satisfactory NPPI (no protein pollution index). Research steps during the development of these diets confirmed that total DNA quantification was the best analytical solution for measuring cross-contamination in these specifically formulated diets based on extensively hydrolysed protein. This approach has also been very efficient in raising the bar of industrial-scale production. The DNA-protein calibrations allowed the level of a particular ancillary protein source in an Anallergenic™ diet sample, the NPPI, to be determined from a measure of its DNA. The observed differences in calibration between the protein sources tested as potential contaminants may be related to either the particular protein source or/and to the way it had been processed by the supplier, for example, the level of grinding and so forth. Further research is warranted to understand these differences. In the absence of a clear consensus on the precise quantity of protein that may trigger signs of allergy in pets, the conformity threshold for cross-contamination tests was taken from a pilot clinical trial in which all dogs with complex or refractory CAFR had clinical improvement in their skin signs when fed the test diet [24]. The threshold for allergens varies between individuals and data from that trial should be regarded as preliminary pending data from a larger number of dogs, preferably including double-blinded rechallenges. Future studies to determine a precise threshold(s) in both species could be useful additional information for the scientific community.

In addition to freedom from cross-contamination with ancillary proteins, the reliability of protein hydrolysate-based diets for elimination trials requires that they have a guaranteed level of hydrolysis and no remaining allergenic fragments [14]. The lower the allergenic potential of a given food in any elimination diet the more reliable the diagnosis of AFR can be [26]. The most common protein allergens range in size from 15 to 40 kDa, although smaller (10 kDa) and larger (70 kDa) molecules can also be immunogenic [27]. Between 3 and 15 kDa, the antigenicity of hydrolysed diets is reduced but allergic reactions are still possible [11]. Studies in children have established that only amino-acid-based and extensively hydrolysed formulas may be considered as truly ‘non-allergenic’ [28] and diets with proteins less than 1 kDa would ensure the greatest chance of reducing allergic reaction [29]. Protein size however is not the only criterion to be considered. Parameters such as the number and location of epitopes and the protein source may be important [27,30], therefore in vivo trials are necessary to confirm the true ‘hypoallergenicity’ of a diet.

Royal Canin Anallergenic^TM^ diets are based on a feather protein hydrolysate, which is a more specific protein hydrolysate than that commonly used in pet food. It was selected primarily for its very high level of hydrolysis but also for its sustainability, being a by-product that does not compete with the human food production chain. Extensive hydrolysis and absence of residual large protein fragments is key to the reliability of the diets [31]. This has been demonstrated in a recent in vitro study on the immune recognition of poultry raw materials in the sera from chicken-allergic dogs and poultry-allergic cats [32]. Classical gel electrophoresis with Coomassie blue staining was insufficiently sensitive to visualize both high molecular weight proteins as potential contaminants and the small oligopeptides and amino acids of the hydrolysate’s proteome. Silver nitrate staining did not allow visualization of a wide enough range of protein sizes. An independent laboratory was therefore commissioned to develop techniques suitable for the analyses required. Proteins were removed from the gels for proteomic analysis using a ‘band-cutting’ technique. This technique was selected because it meant that the whole of the gel at a specific protein size location was sampled with no loss of information. In comparison, a spot picking technique would have allowed good vertical definition but would not be optimally sensitive. Once optimized, the methodologies developed were able to confirm an extensive level of hydrolysis of both the feather protein hydrolysate and the finished Anallergenic™ diets. The importance of this was demonstrated in a recent in vitro study on the immune recognition of poultry raw materials in the sera from chicken-allergic dogs and poultry-allergic cats [32]. The amino acids/oligopeptides observed by thin layer chromatography in all diets were expected because non-anallergenic diets such as Royal Canin^®^ Medium Adult may be supplemented with pure amino-acids to ensure nutritional balance for long-term maintenance feeding. 

No lipid-transfer proteins were detected in the tested Anallergenic™ diets by either electrophoresis or by proteomic analyses. The non-specific lipid-transfer proteins (nsLTPs) are a large protein family found in all land plants. They are considered to be key proteins for plant survival [33] and have been identified as allergens in a wide range of plant foods, including fruits, vegetables, peanuts, tree nuts and cereals [34,35]. The LTP Zea mays 14 (also known as ZmLTP1.6 [33]), a protein of approximately 9 kDa, has been identified as the major corn allergen in humans [23]; it is also suspected of eliciting an allergic response in some corn-allergic pets. It is therefore important that diets formulated for dogs and cats with AFR are made with raw materials free from LTPs, especially if pets are suspected to have a corn allergy. Purified corn starch rather than classical cereal was selected as the carbohydrate source for Royal Canin Anallergenic^TM^ diets for this reason. Purified corn starch is composed of only the purified amylaceous fraction of the corn grain, which is obtained after physical processing of the whole corn kernel has removed the corn steep, germs, fibres and gluten proteins including LTPs. The present studies confirmed the absence of detectable LTPs in Royal Canin Anallergenic™ diets.

The corn protein GBSS-I was identified in the diets tested. GBSS proteins belong to the family of starch granule-associated proteins (SGAPs), which have been described in many carbohydrate sources including wheat, rice, potato tuber, sweet potato, sorghum and banana [36,37,38,39,40,41] and may therefore still be present in some home-made diet recipes. In conjunction with other enzymes GBSS synthesizes amylose in the corn amyloplast—a model has recently been proposed for the catalysis and elongation of sugar chains in the amylose/amylopectin biosynthesis pathway [42]. In a study investigating the clinical relevance of GBSS in humans, serum from patients allergic to corn showed IgE binding to a protein weighing 56 kDa that was present in both corn and rice and that was abundant in the rice endosperm [38]. It seems likely therefore that GBSS detected in the corn starch and finished products studied here had remained trapped in starch granules. 

To the authors’ knowledge, there has been no published description to date of clinical signs triggered by corn GBSS in corn-allergic dogs or cats. In a recent study, Western blot assays performed with serum from dogs with suspected AFR showed that some dogs had circulating IgE antibodies targeting six different carbohydrate proteins detected in three hydrolysed dog foods [13]. Of these proteins, only GBSS-I was of corn origin. IgE-mediated immune recognition of allergenic proteins and diagnostic tests based on this are currently the subject of debate. Although many allergens are pure proteins, some are glycoproteins and some IgEs recognize the glycan part and not the peptidic part of such glycoproteins. The specificity for these glycans is described as being less than that for peptides and the detection of glycan-specific IgEs could result in false positives in IgE-based allergy tests, as has been shown in human medicine. The importance of cross-reactive carbohydrate determinants (CCDs) is discussed in the research literature [43,44]. N-glycan CCD-IgEs may not actually be pathogenic in humans, while a few studies in dogs have shown that adding CCD-blockers when testing for IgEs reduced the number of positive results to some plant allergens in some dogs [43]. More studies are needed to understand the effect of CCD-blockers in this context. Lastly, in a review of the best available evidence for common food allergens in pets, the prevalence of corn-allergic dogs and cats was actually quite low, representing only 4% of all reported food allergens involved in CAFRs in these species [45]. Overall these lines of evidence indicate that further studies are warranted to determine whether GBSS constitutes an actual corn allergen in dogs and cats and whether it is clinically significant in individuals with a known hypersensitivity to corn. It should also be emphasized that the clinical manifestation of AFR depends not only upon the presence of the allergen in the ingested food but also on the quantity and the processing of the raw material, since cooking and processing may reduce IgE sensitization to foods in dogs [46]. 

## 5. Conclusions

AFRs can severely affect a pet’s quality of life. The recommended procedure for the differential diagnosis of non-seasonal pruritus and other suggestive skin signs is an elimination trial that complies with the guidelines of veterinary dermatologists in all details, including trial duration, the optimal choice of elimination diet and dietary re-challenge. The protein composition of hypoallergenic diets for elimination trials and subsequent maintenance feeding is critical. The specific protein source for Royal Canin Anallergenic^TM^ diets for dogs and cats is extensively hydrolysed poultry feather protein. The data presented here confirm that stringent quality control in the manufacturing process (including raw material selection, factory and cleaning adaptations, DNA testing for contaminants) has prevented cross contamination with ancillary protein in both commercial diets. The extensive level of protein hydrolysis in these diets has also been demonstrated. Absence of ancillary protein and extensive proteolysis are both necessary to maximize the clinical reliability of AFR diagnosis and optimize the management of AFR [14]. These results support the previously demonstrated clinical effectiveness of extensively hydrolysed feather protein diets for pets with AFR [47,48,49] and also the strong digestive benefits demonstrated in some dogs suffering from inflammatory bowel disease [50]. Other trials are warranted to confirm their clinical benefits in the management of corn-allergic pets and pets with non-IgE mediated CAFR.

## Figures and Tables

**Table 1:** List of all the samples analysed by protein gel electrophoresis.

Category	Code in Gels	Description	Comments
Complete Diets	RC AFR canine diet AC(a, b, c, d: different production batches)	Royal Canin Anallergenic™ canine diet—the finished product after final coating step	
	RC AFR canine/feline diet BC(a, b, c, d: different production batches)	Royal Canin Anallergenic™ canine or feline diet before final coating step	No palatability enhancers in these samples
	Other AFR diet	Other commercial hypoallergenic dry canine diet	Chicken liver hydrolysate as main protein source
	RC Med Adult 25	Royal Canin^®^ Medium Adult 25	Diet not recommended for AFR but analysed for comparison
Raw materials	Anallergenic™ EHPF	Extensively hydrolysed poultry feather	Anallergenic™ protein source (poultry: chicken, turkey, duck)
	MHPF	Mildly hydrolysed poultry feather	Raw materials not included in Anallergenic™ diets but analysed for comparison
	NHCM	Non-hydrolysed chicken meal	
	Corn flour	Corn flour	
	Feather meal	Feather meal	
	Anallergenic™ corn starch	Anallergenic™ corn starch	

**Table 2:** Correlation between protein level (NPPI %) and DNA concentration for potential ancillary protein sources tested during the calibration.

Processed Animal Protein Used to Experimentally Cross-Contaminate Anallergenic™ Diets at an Inclusion Level (NPPI) of 0.5%	Corresponding DNA Content, µg/g (±0.2 µg/g)
Pork	2.1
Chicken	3.0
Beef	3.1
Duck	4.4

NPPI, no protein pollution index.

## Glossary

AFR	adverse food reaction(s)
CADESI	Canine Atopic Dermatitis Extent and Severity Index
CAFR	cutaneous adverse food reaction(s)
CCD	cross-reactive carbohydrate determinant
FIAD	Food-Induced Allergic Dermatitis
GBSS	granule-bound starch synthase
LTP	lipid-transfer proteins
nsLTP	non-specific lipid-transfer protein
MALDI	Matrix Assisted Laser Desorption Ionisation
NPPI	no protein pollution index
TOF	Time Of Flight detector
